# Editorial Announcement

**Published:** 1915-06

**Authors:** 


### EDITORIAL ANNOUNCEMENT.

---

Owing to pressure of other duties, Hon. Henry W. Hill has been unable to prepare an article on the anti-narcotic laws for this issue.

---

Between now and fall, we shall have room for about six additional original articles of average length—addresses, etc., not desired.

